# Complete genome sequence of the thermophilic sulfur-reducer *Hippea maritima* type strain (MH_2_^T^)

**DOI:** 10.4056/sigs.1814460

**Published:** 2011-06-30

**Authors:** Marcel Huntemann, Megan Lu, Matt Nolan, Alla Lapidus, Susan Lucas, Nancy Hammon, Shweta Deshpande, Jan-Fang Cheng, Roxanne Tapia, Cliff Han, Lynne Goodwin, Sam Pitluck, Konstantinos Liolios, Ioanna Pagani, Natalia Ivanova, Galina Ovchinikova, Amrita Pati, Amy Chen, Krishna Palaniappan, Miriam Land, Loren Hauser, Cynthia D. Jeffries, John C. Detter, Evelyne-Marie Brambilla, Manfred Rohde, Stefan Spring, Markus Göker, Tanja Woyke, James Bristow, Jonathan A. Eisen, Victor Markowitz, Philip Hugenholtz, Nikos C. Kyrpides, Hans-Peter Klenk, Konstantinos Mavromatis

**Affiliations:** 1 Biological Data Management and Technology Center, Lawrence Berkeley National Laboratory, Berkeley, California, USA; 2 DOE Joint Genome Institute, Walnut Creek, California, USA; 3 Los Alamos National Laboratory, Bioscience Division, Los Alamos, New Mexico, USA; 4 Oak Ridge National Laboratory, Oak Ridge, Tennessee, USA; 5 DSMZ - German Collection of Microorganisms and Cell Cultures GmbH, Braunschweig, Germany; 6 HZI – Helmholtz Centre for Infection Research, Braunschweig, Germany; 7 University of California Davis Genome Center, Davis, California, USA; 8 Australian Centre for Ecogenomics, School of Chemistry and Molecular Biosciences, The University of Queensland, Brisbane, Australia

**Keywords:** anaerobic, motile, rod-shaped, Gram-negative, marine, moderately thermophilic, sulfur-reducer, *Desulfurellaceae*, GEBA

## Abstract

*Hippea maritima* (Miroshnichenko *et al*. 1999) is the type species of the genus *Hippea,* which belongs to the family *Desulfurellaceae* within the class *Deltaproteobacteria*. The anaerobic, moderately thermophilic marine sulfur-reducer was first isolated from shallow-water hot vents in Matipur Harbor, Papua New Guinea. *H. maritima* was of interest for genome sequencing because of its isolated phylogenetic location, as a distant next neighbor of the genus *Desulfurella*. Strain MH_2_^T^ is the first type strain from the order *Desulfurellales* with a completely sequenced genome. The 1,694,430 bp long linear genome with its 1,723 protein-coding and 57 RNA genes consists of one circular chromosome and is a part of the *** G****enomic* *** E****ncyclopedia of* *** B****acteria and* *** A****rchaea * project.

## Introduction

Strain MH_2_^T^ (DSM 10411 = ATCC 700847) is the type strain of the species *Hippea maritima*, which is the type species of its genus *Hippea* [1]. The genus currently contains no other validly named species [2], but two other strains belonging to the species were isolated from shallow-water hot vents in New Zealand and Papua New Guinea [1]. The type strain was isolated during a cruise of the Russian scientific vessel *A. Nesmeyanov* through shallow-water hot vents of the south-western Pacific Ocean, environments that are typical for anaerobic, thermophilic, sulfur-reducing bacteria [1]. The genus is named after the German microbiologist Hans Hippe, in recognition of his significant contribution to the characterization of novel, obligately anaerobic prokaryotes and the understanding of their physiology. The species epithet is derived from the Latin word *maritima* (inhabiting marine environments) [2]. Here we present a summary classification and a set of features for *H. maritima* strain MH_2_^T^, together with the description of the complete genomic sequencing and annotation.

## Classification and features

A representative genomic 16S rRNA sequence of strain MH_2_^T^ was compared using NCBI BLAST under default settings (e.g., considering only the high-scoring segment pairs (HSPs) from the best 250 hits) with the most recent release of the Greengenes database [3] and the relative frequencies, of taxa and keywords (reduced to their stem [4]) were determined, weighted by BLAST scores. The most frequently occurring genera were *Desulfurella* (38.7%), *Desulfovibrio* (15.2%), *Deferribacter* (10.8%), *Thermotoga* (10.8%) and *Hippea* (8.6%) (44 hits in total). Regarding the single hit to sequences from members of the species, the average identity within HSPs was 99.9%, whereas the average coverage by HSPs was 82.7%. Among all other species, the one yielding the highest score was *Desulfurella multipotens*, which corresponded to an identity of 89.6% and an HSP coverage of 82.6%. (Note that the Greengenes database uses the INSDC (= EMBL/NCBI/DDBJ) annotation, which is not an authoritative source for nomenclature or classification.) The highest-scoring environmental sequence was AF232926 ('United Kingdom: Montserrat geothermal springs clone MS10 proteobacterium'), which showed an identity of 88.9% and a HSP coverage of 73.0%. The most frequently occurring keywords within the labels of environmental samples which yielded hits were 'microbi' (5.0%), 'spring' (2.9%), 'sediment' (2.4%), 'soil' (2.3%) and 'industri' (2.2%) (206 hits in total). Environmental samples which yielded hits of a higher score than the highest scoring species were not found.

**Figure 1:**
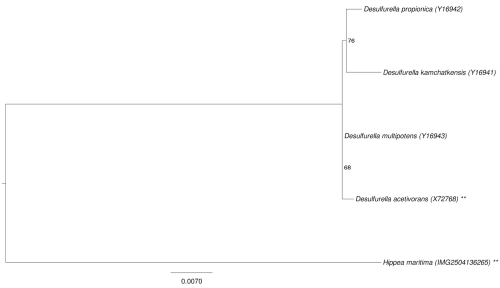
Phylogenetic tree highlighting the position of *H. maritima* relative to the other type strains within the family *Desulfurellaceae*. The tree was inferred from 1,526 aligned characters [5,6] of the 16S rRNA gene sequence under the maximum likelihood criterion [7] and rooted in accordance to the current taxonomy. The branches are scaled in terms of the expected number of substitutions per site. Numbers next to bifurcations are support values from 700 bootstrap replicates [8] if larger than 60%. Lineages with type strain genome sequencing projects registered in GOLD [9] are shown with an asterisk, those also listed as 'Complete and Published' with two asterisks.

The 16S rRNA based tree in Figure 1 shows the phylogenetic neighborhood of *H. maritima*. The sequence of the two identical 16S rRNA genes differs by one nucleotide from the previously published 16S rRNA sequence (Y18292).

**Figure 2:**
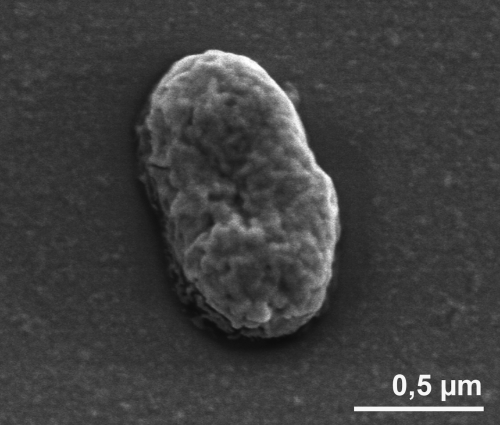
Scanning electron micrograph of *H. maritima* MH_2_^T^

The cells of *H. maritima* are short rods ranging from 1-3 x 0.4–0.8 µm (Figure 2 and Table 1) that occur singly or in pairs [1]. *H. maritima* is motile by one polar flagellum [1] (not visible in Figure 2). Colonies are whitish-gray with diameters up to 0.5 mm [1]. *H. maritima* cultures require 2.5-3% NaCl and 0.02% (w/v) yeast extract for growth [1]. The temperature range for growth is between 40°C and 65°C, with an optimum at 52–54°C [1]. Growth was observed over a pH range of 5.7 to 6.5 with an optimum around 6.0 [1].

**Table 1:** Classification and general features of *H. maritima* MH_2_^T^ according to the MIGS recommendations [10].

**MIGS ID**	**Property**	**Term**	**Evidence code**
	Current classification	Domain *Bacteria*	TAS [11]
		Phylum *Proteobacteria*	TAS [12]
		Class *Deltaproteobacteria*	TAS [13,14]
		Order *Desulfurellales*	TAS [13,14]
		Family *Desulfurellaceae*	TAS [14,15]
		Genus *Hippea*	TAS [1]
		Species *Hippea maritima*	TAS [1]
		Type strain MH_2_	TAS [1]
	Gram stain	negative	TAS [1]
	Cell shape	short rods	TAS [1]
	Motility	motile, one polar flagellum	TAS [1]
	Sporulation	never observed	TAS [1]
	Temperature range	40-56°C	TAS [1]
	Optimum temperature	52-54°C	TAS [1]
	Salinity	2.5-3% NaCl	TAS [1]
MIGS-22	Oxygen requirement	anaerobic	TAS [1]
	Carbon source	saturated fatty acids (stearate, palmitate)	TAS [1]
	Energy metabolism	acetate, long-chain saturated fatty acids; lithotrophic growth with H_2_ and S_0_	TAS [1]
MIGS-6	Habitat	submarine hot vents	TAS [1]
MIGS-15	Biotic relationship	free-living	NAS
MIGS-14	Pathogenicity	none	NAS
	Biosafety level	1	TAS [16]
	Isolation	hot vents in tidal zone	TAS [1]
MIGS-4	Geographic location	Matupi Harbour, Papua New Guinea	TAS [1]
MIGS-5	Sample collection time	1999	TAS [1,17]
MIGS-4.1	Latitude	-4.23	NAS
MIGS-4.2	Longitude	152.2	NAS
MIGS-4.3	Depth	not reported	
MIGS-4.4	Altitude	approximately sea level	NAS

Evidence codes - IDA: Inferred from Direct Assay (first time in publication); TAS: Traceable Author Statement (i.e., a direct report exists in the literature); NAS: Non-traceable Author Statement (i.e., not directly observed for the living, isolated sample, but based on a generally accepted property for the species, or anecdotal evidence). These evidence codes are from of the Gene Ontology project [18]. If the evidence code is IDA, the property was directly observed by one of the authors or an expert mentioned in the acknowledgements.

All *H. maritima* strains can grow on molecular hydrogen, acetate, and saturated fatty acids and require elemental sulfur as the only known electron acceptor [1]. Strain MH_3_, isolated from Matupi Harbor, was the only *H. maritima* strain growing on ethanol in the presence of elemental sulfur [1]. Fumarate supported only weak growth for all three known strains [1], whereas formate, propionate, butyrate, pyruvate, lactate, succinate, glucose, starch, peptone, methanol did not support growth [1]. CO_2_ and H_2_S were the only detected end products [1].

### Chemotaxonomy

No chemotaxonomical data were reported in the initial description of the organism [1] nor elsewhere, subsequently.

## Genome sequencing and annotation

### Genome project history

This organism was selected for sequencing on the basis of its phylogenetic position [19], and is part of the *** G****enomic* *** E****ncyclopedia of* *** B****acteria and* *** A****rchaea * project [20]. The genome project is deposited in the Genomes On Line Database [9] and the complete genome sequence is deposited in GenBank. Sequencing, finishing and annotation were performed by the DOE Joint Genome Institute (JGI). A summary of the project information is shown in Table 2.

**Table 2:** Genome sequencing project information

**MIGS ID**	**Property**	**Term**
MIGS-31	Finishing quality	Finished
MIGS-28	Libraries used	Three genomic libraries: one 454 pyrosequence standard library, one 454 PE library (7.3 kb insert size), one Illumina library
MIGS-29	Sequencing platforms	Illumina GAii, 454 GS FLX Titanium
MIGS-31.2	Sequencing coverage	1,213 × Illumina; 29.6 × pyrosequence
MIGS-30	Assemblers	Newbler version 2.3, Velvet version 0.7.63, phrap version SPS-4.24
MIGS-32	Gene calling method	Prodigal 1.4, GenePRIMP
	INSDC ID	CP002606
	Genbank Date of Release	March 29, 2011
	GOLD ID	Gc01705
	NCBI project ID	48195
	Database: IMG-GEBA	2504136000
MIGS-13	Source material identifier	DSM 10411
	Project relevance	Tree of Life, GEBA

### Growth conditions and DNA isolation

*H. maritima* MH_2_^T^, DSM 10411, was grown anaerobically in medium 554 (HIPPEA medium) [21] at 55°C. DNA was isolated from 0.5-1 g of cell paste using Jetflex Genomic DNA Purification Kit (GENOMED 600100) following the standard protocol as recommended by the manufacturer with the following modification to improve cell lysis: additional 20µl lysozyme (100mg/µl) and 10µl mutalysin were used for 30 min incubation at 37°C, followed by three hours incubation at 58°C with 20µl proteinase K. DNA is available through the DNA Bank Network [22].

### Genome sequencing and assembly

The genome was sequenced using a combination of Illumina and 454 sequencing platforms. All general aspects of library construction and sequencing can be found at the JGI website [23]. Pyrosequencing reads were assembled using the Newbler assembler (Roche). The initial Newbler assembly, consisting of 70 contigs in one scaffold, was converted into a phrap [24] assembly by making fake reads from the consensus to collect the read pairs in the 454 paired end library. Illumina GAii sequencing data (4,403.8 Mb) was assembled with Velvet [25] and the consensus sequences were shredded into 1.5 kb overlapped fake reads and assembled together with the 454 data. The 454 draft assembly was based on 66.2 Mb 454 draft data and all of the 454 paired end data. Newbler parameters are -consed -a 50 -l 350 -g -m -ml 20. The Phred/Phrap/Consed software package [24] was used for sequence assembly and quality assessment in the subsequent finishing process. After the shotgun stage, reads were assembled with parallel phrap (High Performance Software, LLC). Possible mis-assemblies were corrected with gapResolution [23], Dupfinisher [26], or sequencing cloned bridging PCR fragments with subcloning or transposon bombing (Epicentre Biotechnologies, Madison, WI). Gaps between contigs were closed by editing in Consed, by PCR and by Bubble PCR primer walks (J.-F. Chang, unpublished). A total of 357 additional reactions and one shatter library were necessary to close gaps and to raise the quality of the finished sequence. Illumina reads were also used to correct potential base errors and increase consensus quality using a software Polisher developed at JGI [27]. The error rate of the completed genome sequence is less than 1 in 100,000. Together, the combination of the Illumina and 454 sequencing platforms provided 1,241.6 × coverage of the genome. The final assembly contained 112,403 pyrosequence and 57,283,044 Illumina reads.

### Genome annotation

Genes were identified using Prodigal [28] as part of the Oak Ridge National Laboratory genome annotation pipeline, followed by a round of manual curation using the JGI GenePRIMP pipeline [29]. The predicted CDSs were translated and used to search the National Center for Biotechnology Information (NCBI) non-redundant database, UniProt, TIGR-Fam, Pfam, PRIAM, KEGG, COG, and InterPro databases. Additional gene prediction analysis and functional annotation were performed within the Integrated Microbial Genomes - Expert Review (IMG-ER) platform [30].

## Genome properties

**Figure 3:**
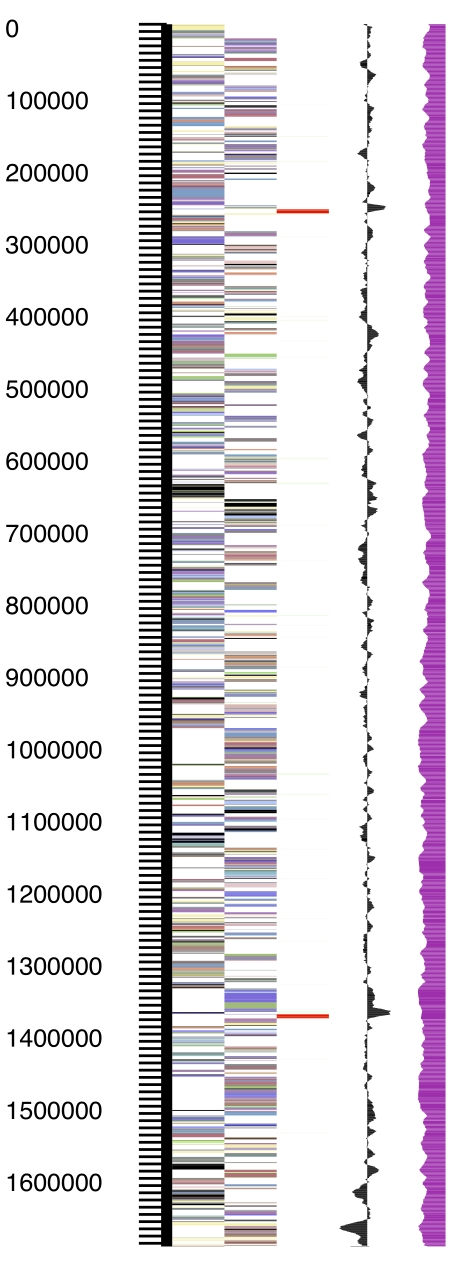
Graphical map of the linear chromosome. From left to right: Genes on forward strand (color by COG categories), Genes on reverse strand (color by COG categories), RNA genes (tRNAs green, rRNAs red, other RNAs black), GC content, GC skew.

The genome consists of a 1,694,430 bp long linear chromosome with a G+C content of 37.5% (Table 3 and Figure 3). Of the 1,780 genes predicted, 1,723 were protein-coding genes, and 57 RNAs; 46 pseudogenes were also identified. The majority of the protein-coding genes (76.4%) were assigned with a putative function while the remaining ones were annotated as hypothetical proteins. The distribution of genes into COGs functional categories is presented in Table 4.

**Table 3:** Genome Statistics

**Attribute**	**Value**	**% of Total**
Genome size (bp)	1,694,430	100.00%
DNA coding region (bp)	1,580,424	93.27%
DNA G+C content (bp)	634,975	37.47%
Number of replicons	1	
Extrachromosomal elements	0	
Total genes	1,780	100.00%
RNA genes	57	3.20%
rRNA operons	2	
Protein-coding genes	1,723	96.80%
Pseudo genes	46	2.58%
Genes with function prediction	1,360	76.40%
Genes in paralog clusters	182	10.22%
Genes assigned to COGs	1,414	79.44%
Genes assigned Pfam domains	1,485	83.43%
Genes with signal peptides	261	14.66%
Genes with transmembrane helices	423	23.76%
CRISPR repeats	0	

**Table 4:** Number of genes associated with the general COG functional categories

**Code**	**value**	**% age**	**Description**
J	133	8.5	Translation, ribosomal structure and biogenesis
A	0	0.0	RNA processing and modification
K	45	2.9	Transcription
L	119	7.6	Replication, recombination and repair
B	0	0.0	Chromatin structure and dynamics
D	19	1.2	Cell cycle control, cell division, chromosome partitioning
Y	0	0.0	Nuclear structure
V	11	0.7	Defense mechanisms
T	78	5.0	Signal transduction mechanisms
M	110	7.1	Cell wall/membrane/envelope biogenesis
N	69	4.4	Cell motility
Z	0	0.0	Cytoskeleton
W	0	0.0	Extracellular structures
U	59	3.8	Intracellular trafficking, secretion, and vesicular transport
O	68	4.4	Posttranslational modification, protein turnover, chaperones
C	107	6.9	Energy production and conversion
G	62	4.0	Carbohydrate transport and metabolism
E	147	9.4	Amino acid transport and metabolism
F	46	3.0	Nucleotide transport and metabolism
H	108	6.9	Coenzyme transport and metabolism
I	52	3.3	Lipid transport and metabolism
P	66	4.2	Inorganic ion transport and metabolism
Q	22	1.4	Secondary metabolites biosynthesis, transport and catabolism
R	146	9.4	General function prediction only
S	93	6.0	Function unknown
-	366	20.6	Not in COGs
